# Accounting for individual differences in speed in the discretized signed residual time model

**DOI:** 10.1111/bmsp.12223

**Published:** 2020-12-22

**Authors:** Jesper Tijmstra, Maria Bolsinova

**Affiliations:** ^1^ Department of Methodology and Statistics Faculty of Social Sciences Tilburg University The Netherlands

**Keywords:** Signed residual time model, Response time, Response speed, Item response theory, Ability measurement

## Abstract

With advances in computerized tests, it has become commonplace to register not just the accuracy of the responses provided to the items, but also the response time. The idea that for each response both response accuracy and response time are indicative of ability has explicitly been incorporated in the signed residual time (SRT) model (Maris & van der Maas, 2012, Psychometrika, *77*, 615–633), which assumes that fast correct responses are indicative of a higher level of ability than slow correct responses. While the SRT model allows one to gain more information about ability than is possible based on considering only response accuracy, measurement may be confounded if persons show differences in their response speed that cannot be explained by ability, for example due to differences in response caution. In this paper we propose an adapted version of the SRT model that makes it possible to model person differences in overall speed, while maintaining the idea of the SRT model that the speed at which individual responses are given may be indicative of ability. We propose a two‐dimensional SRT model that considers dichotomized response time, which allows one to model differences between fast and slow responses. The model includes both an ability and a speed parameter, and allows one to correct the estimates of ability for possible differences in overall speed. The performance of the model is evaluated through simulation, and the relevance of including the speed parameter is studied in the context of an empirical example from formative educational assessment.

## 1 Introduction

With advances in computerized testing for ability measurement in recent years it has become more and more common in testing settings for not only the accuracy of the responses provided but also their response time to be available. While standard item response theory (IRT; see, for example, Hambleton, Swaminathan, & Rogers, 1991; van der Linden & Hambleton, 2013) models only make use of information available on response accuracy (RA) for the estimation of ability and hence only consider whether (or the extent to which) an answer is correct, a variety of statistical models have been proposed that also take response times (RTs) into account.

One of the most commonly used statistical frameworks for making use of RTs for the estimation of ability is the hierarchical modelling framework proposed by van der Linden (2007). In this framework, RT and RA are both treated as random variables and are jointly modelled. On the person side, the RAs are taken to be explained by a latent ability parameter, while the RTs are taken to be explained by a latent speed parameter. This latent speed parameter represents *response speed* (rather than, for example, cognitive speed), as it captures the speed at which a respondent provides responses on the test. Since under the hierarchical model in the population of persons response speed and ability may be correlated, the model can make use of collateral information about ability contained in the RTs to improve the precision of measurement (van der Linden, Klein Entink, & Fox, 2010). However, under the hierarchical model all relevant information about ability available in the RTs is taken to be captured by the latent speed parameter, meaning that, conditional on speed, RTs are taken not to contain any relevant collateral information about ability (Bolsinova & Tijmstra, 2018). Consequently, the extent to which considering RTs improves the precision of measurement when using the hierarchical model is limited by the extent to which speed and ability are correlated in the population (Ranger, 2013), and in practice correlations close to zero are not uncommon (see, for example, Bolsinova, De Boeck, & Tijmstra, 2017; Bolsinova, Tijmstra, & Molenaar, 2017; van der Linden, Scrams, & Schnipke, 1999).

Rather than assuming only someone's overall speed to be informative of ability, it may be plausible that the RTs of each specific response can be relevant to the estimation of ability. For example, it may be reasonable that on some tests giving correct answers while taking little time may be indicative of a higher level of ability than only giving correct answers after having taken a lot of time. The idea that for each response both RA and RT are indicative of ability has explicitly been incorporated in the signed residual time (SRT) model (Maris & van der Maas, 2012), which directly incorporates RT into the scoring rule for the measurement of ability. The marginal model for RA is a two‐parameter logistic model (2PLM), and hence ability as measured using the SRT model (provided it is an appropriate model for the data) will fully match ability as measured using only the RA data (using a 2PLM). However, while the measured ability is the same in the 2PLM and the SRT model, the latter increases the precision of measurement by making use of collateral information in the RT of each response.

One important characteristic of the SRT model is that it is a normative model, in the sense that its scoring rule needs to be communicated to all respondents such that they can perform optimally on the test. That is, in applications where one wants to make use of an SRT model, one needs to be explicit about the way in the time taken before responding affects the score that is obtained on the item: correct responses receive more credit when they are made fast rather than slowly, while fast careless responding is discouraged by penalizing fast incorrect responses more heavily than slow incorrect responses (Maris & van der Maas, 2012). Since the scoring rule is communicated, respondents are assumed to optimize their test‐taking behaviour in terms of the response speed that they adopt when answering the items.

It may be noted that there are important conceptual and statistical differences as well as similarities between the SRT model and the hierarchical model. For both models, the marginal model for RA is a simple IRT model, meaning that when either of these models applies, ability as estimated by the joint model for RA and RT is identical to the ability that is estimated when only the RA data are considered (using the correct measurement model). Hence, in both models the nature of the estimated ability is not altered by the inclusion of RTs in the model. Both models make use of collateral information available in the RT data to improve the precision with which this ability is estimated, but they differ in the way in which RTs are incorporated. In the case of the hierarchical model, RTs are not directly linked to ability, but rather are taken to be fully explained by a latent speed variable that in turn provides collateral information for the estimation of ability. Thus, although speed and ability may be correlated, the model still assumes there to be variability in speed conditional on the latent variable (provided the correlation is not 1 or −1) and respondents of the same ability level may differ in their response speed. In contrast, the SRT model does not include a latent speed parameter, and assumes that, conditional on ability, all respondents will operate at exactly the same speed. That is, the expected response time on a particular item is the same for all persons of a particular ability level, from which it follows that the model assumes that respondents of the same ability level all operate at the same speed.

Although the SRT model is a normative model, it can be considered unlikely that respondents fully succeed in optimizing their response speed, meaning that individual differences in RTs may remain that cannot be accounted for under the model. The many applications of the hierarchical model tell us that there is often a positive manifold for the RTs, suggesting that some respondents consistently respond at a higher speed than others. Since in practice positive (e.g., van der Linden, 2007; Meng, Tao, & Chang, 2015), negative (e.g., Klein Entink, Fox, & van der Linden, 2009; van der Linden & Guo, 2008), and approximately zero (e.g., Bolsinova, Tijmstra, et al., 2017; van der Linden et al., 1999) values have been reported for the estimated correlation between speed and ability, it is clear that these individual differences in response speed generally cannot be fully accounted for by considering ability. While settings in which one aims to use the SRT model may differ from those where the hierarchical model has been considered (since in those settings one would explicitly communicate how RT affects the item scores), it may be plausible that such individual differences in response speed will at least partly remain, even after respondents have become familiar with the scoring rule. Individual differences in risk aversion and risk seeking have been extensively studied in the psychological literature (e.g., see Eisenberg, Baron, & Seligman, 1998; Horvath & Zuckerman, 1993; Lerner & Keltner, 2001; Zuckerman & Kuhlman, 2000), and it seems natural to assume that such tendencies will to some extent also play a role in settings where the SRT model is applied, leading to suboptimal test‐taking behaviour: risk‐seeking respondents may be tempted to respond too quickly, while risk‐averse respondents may take longer to respond than is optimal for their expected item score.

When person differences in response speed are present that are not accounted for under the SRT model, two statistical issues arise: first, model misfit will be present; and second, person estimates will be biased. A consequence of model misfit may be that estimated item parameters are affected, for example (if included in the model) item discrimination parameters might be underestimated due to the model being less able to explain person differences in observed performance. Bias in the person estimates can be expected both for respondents who display suboptimal responding behaviour in terms of their response speed (resulting in their ability being underestimated) and for respondents who behave optimally (whose ability may be overestimated since the assessment of their ability relative to that of their peers benefits from the fact that suboptimally behaving peers are underperforming). These two issues make it important to exclude the possible presence of unmodelled person differences in response speed when using the SRT model, or, if such differences are present, to incorporate such differences in the statistical model.

In this paper we propose an IRT model that – similar to the SRT model – measures ability based on both RA and RT, but unlike the SRT model also takes into account that persons may differ in their response speed. To facilitate statistical modelling, responses are categorized as being either fast or slow, resulting in four possible combinations of RA and RT. The proposed two‐dimensional discretized SRT model maintains the core idea of the SRT model, as it assigns more credit to fast correct responses than to slow correct responses and assigns less credit to fast incorrect responses than to slow incorrect responses. Like the SRT model, in the proposed model performance is explained on the person side by an ability parameter. However, the model includes an additional person parameter, which explains the relative frequency with which fast responses are given (and hence captures a person's tendency to give fast rather than slow responses). As such, it can account for person differences in performance that are not due to differences in ability but rather due to differences in a response tendency (e.g., response caution), and can correct the ability estimates for these person differences that may otherwise confound measurement. In this way, the model is designed to combine the advantages of the SRT model (i.e., incorporating RT information directly into the scoring rule) and of the hierarchical model (i.e., taking between‐person differences in response speed into account), while avoiding their limitations as discussed. Furthermore, the latent variable included may also provide test takers and test administrators with additional relevant information, since it provides insight into the extent to which a test taker deviated from the model‐implied optimal response speed. This could be useful feedback for improving future test‐taking performance, and it could also be of substantive interest in its own right, if such a latent variable relates to psychological constructs such as risk seeking or risk avoidance.

The structure of the paper is the following. In Section 2 we describe the SRT model. Section 3 presents a motivating example from formative educational assessment, where empirical evidence is found that there may be relevant person differences in response speed that are not accounted for by the SRT model. Section 4 presents a two‐dimensional discretized version of the SRT model that accounts for person differences in response speed. In Section 5 we present a simulation study that illustrates the importance of correcting for person differences in speed, where we consider the bias and variance in the estimates of the ability parameters if person differences in speed are or are not taken into account. Section 6 revisits the empirical example, illustrates the applicability of the two‐dimensional discretized SRT model and compares its results to those obtained using a variety of alternative statistical models. The paper concludes with a discussion.

## 2 Signed residual time model

The SRT model was proposed by Maris and van der Maas (2012), and uses the following scoring rule:

(1) $$S_{i}=\left(2X_{i}-1\right)\left(d-T_{i}\right),$$

where *S_i_* is the item score (with realizations *s_pi_* for each person *p*), *X_i_* is the binary RA (1 for correct, 0 for incorrect), *T_i_* is continuous RT, *d* is the imposed item time limit, and index *i* refers to a particular item. The model assumes there to be an item time limit, which in this version of the model is taken to be the same for all items. The scoring rule thus entails that the time that is left before the deadline (i.e., residual time) is either added to the total score if the response is correct, or subtracted from it if the response is incorrect. The idea of the scoring rule is to give more credit to fast correct responses than to slow correct responses, and also to discourage fast careless responding by punishing fast incorrect responses more heavily than slow incorrect responses. The SRT model is derived from the sufficiency of the person total score $s_{p+}=\sum_{i}s_{\mathrm{pi}}$ for the ability of person *p*, the sufficiency of the item total score $s_{i+}=\sum_{p}s_{\mathrm{pi}}$ for the difficulty of item *i*, and conditional independence of the item scores given the latent variable.

In the SRT model the distribution of item RA and RT given ability (denoted by θ) is written as

(2) $$f\left(X_{i},T_{i}|\theta \right)=\frac{\left(\theta -\delta_{i}\right)\exp\left(\left(2X_{\mathrm{pi}}-1\right)\left(d-T_{i}\right)\left(\theta -\delta_{i}\right)\right)}{\exp\left(\theta -\delta_{i}\right)-\exp\left(-\left(\theta -\delta_{i}\right)\right)},$$

where δ*_i_* is the difficulty of item *i*. The SRT model has been generalized by van Rijn and Ali (2018), who introduced an item discrimination parameter, denoted by α_i_> 0

(3) $$f\left(X_{i},T_{i}|\theta \right)=\frac{\alpha_{i}\left(\theta -\delta_{i}\right)\exp\left(\left(2X_{\mathrm{pi}}-1\right)\left(d-T_{i}\right)\alpha_{i}\left(\theta -\delta_{i}\right)\right)}{\exp\left(\theta -\delta_{i}\right)-\exp\left(-\left(\theta -\delta_{i}\right)\right)}$$

By including a discrimination parameter in the model, it becomes possible to account for differences between the items in terms of the strength of the relationship between the item score and ability.

## 3 Motivating example

To illustrate the relevance of including a secondary dimension in the SRT model capturing person differences in response speed, we present an example from educational measurement. We use data from Math Garden (Klinkenberg, Straatemeier, & van der Maas, 2011; Straatemeier, 2014), which is an online adaptive practice system for arithmetic in which children can do exercises covering several domains of mathematics, such as addition, subtraction and multiplication. In this study we will consider data for the single‐digit multiplication items (e.g., ‘3 × 6=?’ and ‘8 × 1=?’). The items were all open ended, and for each item the time limit was 20 seconds, with RT being recorded in milliseconds. The SRT scoring rule is built into the system of Math Garden, and it is explicitly communicated to the students by showing them a number of coins which decreases linearly with time, representing the reward that is gained for providing a correct response or lost if an incorrect response is produced. The system does not allow for RTs above the time limit.

For this application, data obtained between 1 September and 1 October 2015 were used. Only data from persons who produced at least 15 responses on a single day were used, and only data obtained on the day on which they had produced the largest number of responses were considered. This was done to ensure that both speed and ability are likely to be stable across the responses of that person. Items with at least 1,000 responses were included. The data set selected included 31 items and 3,099 respondents. Not all items were administered to all persons, with the total proportion of missing responses equal to .57. On average 13 responses were observed per person, and 1,329 responses were observed per item.

For these data we examined whether RTs on different items are generally positively correlated with each other, that is, whether a positive manifold is observed for the RTs. The correlations between the RTs on different items were computed, and the eigenvalues of the correlation matrix were evaluated. The first eigenvalue was equal to 10.26, indicating that the 31 items share a relatively large amount of common variance, which can be interpreted as there being individual differences between persons in whether they tend to give fast or slow responses to most items. Next, we examined whether such a positive manifold of the RTs can be explained by the SRT model, or whether such individual differences between persons in response speed cannot be accounted for under the model.

The SRT model with item‐specific discrimination parameters was fitted to the data using a Gibbs sampler.^1^ Using samples from the posterior distribution of the model parameters, 500 replicated data sets were generated. In each of these data sets the first eigenvalue of the correlation matrix of the RTs was computed, which resulted in 500 samples from the posterior predictive distribution of this statistic. The 2.5th and the 97.5th percentiles of this distribution were equal to 2.42 and 2.76, respectively. That is, in the posterior predictive distribution under the SRT model RTs are not nearly as strongly related to each other as they are in the observed data. Hence, the SRT model cannot sufficiently account for these observed associations. Thus, even though the scoring rule was explicitly communicated to the respondents, they do not all follow it in the same way: some respondents tend to give faster responses than would be expected based on the SRT model to most items, regardless of the accuracy, while others tend to respond more slowly than expected. As discussed in Section 1, these unmodelled differences can be expected to result in model misfit and a confounding of measurement of ability, highlighting the need to include an additional person parameter in the model in order to account for person differences in response speed.

## 4 A two‐dimensional discretized signed residual time model

While developing a direct extension of the SRT model that includes a latent response speed parameter is not straightforward, one can make use of a variety of IRT‐based modelling options if one discretizes continuous RT, which is the direction that will be pursued in this paper. An adapted version of the SRT model has been considered by Coomans, Hofman, Brinkhuis, van der Maas, and Maris (2016), who assign discrete item scores based on whether the response was correct or incorrect, and based on whether it was fast or slow. Let *S_i_* be the discrete item score of item *i*, which can be assigned in the following way:

(4) $$S_{i}=\left\{\begin{array}{c} 0,\;\text{if}\;X_{i}=0,T_{i}^{*}=1,\\ 1,\;\text{if}\;X_{i}=0,T_{i}^{*}=0,\\ 2,\;\text{if}\;X_{i}=1,T_{i}^{*}=0,\\ 3,\;\text{if}\;X_{i}=1,T_{i}^{*}=1, \end{array}\right.$$

where $T_{i}^{*}$ is a dichotomous variable that takes the value 1 if the response is fast and 0 otherwise. In line with the SRT model, this means that the least credit is assigned to a fast and incorrect response, followed by a slow incorrect response, a slow correct response, and a fast correct response. Since the model makes use of discretized RT and considers discrete rather than continuous item scores, this adapted version of the SRT model is here labelled the `discretized SRT model'.

**Figure 1:**
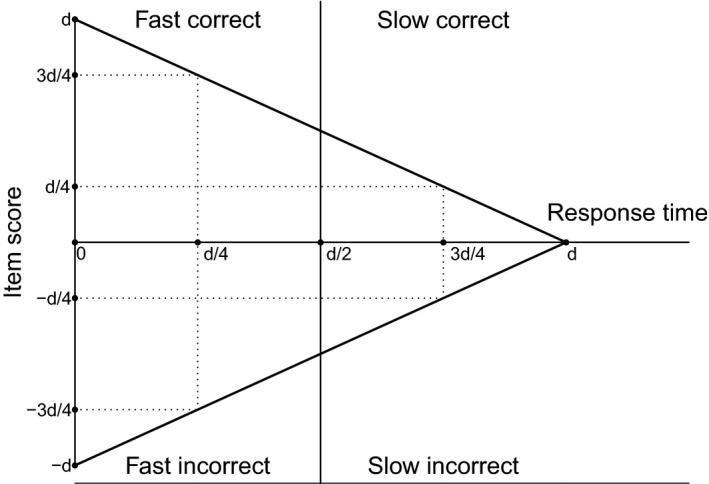
Illustration of the scoring rule that is implied by the continuous signed residual time model for the discretized signed residual time model when its discretization is at half deadline, where *d* denotes the deadline and where the vertical line indicates the split into fast and slow responses. The two unbroken converging lines represent the score under the continuous signed residual time model, while the broken lines represent the implied scores for each of the four response outcomes under the discretized signed residual time model.

While in principle any dichotomization of *T_i_* can be considered, for the discretized SRT model the most natural approach is to set the boundary between fast and slow responses at exactly half the item deadline. Under this specification (and assuming RTs to be uniformly distributed), the scoring rule of the discretized SRT model follows directly from the scoring rule of the continuous SRT model. This is illustrated graphically in Figure 1, where it is shown that setting the boundary at half the item deadline entails that if the continuous SRT model holds, the average score distance between each of the four ordered response outcomes is the same. That is, in that case the SRT model implies that the score difference between a fast incorrect and a slow incorrect response, between a slow incorrect and a slow correct response, and between a slow correct and a fast correct response is the same (i.e., has a value of ½ d). For any other dichotomization, the differences between the ordered response options that are implied by the continuous SRT model will not be of equal magnitude, and assigning a 0, 1, 2, 3 coding would result in a scoring rule that is not aligned with that of the continuous SRT model. For this reason the default dichotomization that will be considered in this paper is based on a boundary at half the item deadline.

Using the discretized SRT model allows one to study relevant differences between fast and slow responses. The objective of Coomans et al. (2016) was to obtain simple sufficient statistics for both the items and the persons, and hence they considered a model where items and persons are each characterized by a single parameter:

(5) $$\mathrm{Pr}\left(S_{i}=s|\theta \right)=\frac{\exp(s\left(\theta -\delta_{i}\right))}{\sum_{r=0}^{3}\exp(r\left(\theta -\delta_{i}\right))},$$

where *s* is a possible realization of the random variable *S_i_*. Under this model, for any person *j*, is $\sum_{i}s_{\mathrm{ji}}$ a sufficient statistic for θ, and for any item *i*, $\sum_{i}s_{\mathrm{ji}}$ is a sufficient statistic for δ*_i_*, where *s_ji_* is the observed score of person *j* on item *i*.

By discretizing the item scores, the items become polytomously scored, and it may make sense to make use of standard polytomous IRT for modelling these item scores. When contrasted with common polytomous IRT models, the model in equation (5) is rather restrictive, since for each item it contains only a single item parameter. Instead it may make sense to consider more flexible models to better capture the structure in the data. If on the person side one wants to preserve sufficiency of $\sum_{i}s_{\mathrm{ji}}$, one can consider a partial credit model (Masters, 1982) for the item scores:

(6) $$\mathrm{Pr}\left(S_{i}=s|\theta \right)=\frac{\exp(s\theta +\beta_{\mathrm{is}})}{\sum_{r=0}^{3}\exp(r\theta +\beta_{\mathrm{ir}})}.$$

where $\beta_{i0}\equiv 0$, and $\beta_{i1},\beta_{i2},\beta_{i3}$ are item category intercept parameters. This model is more general than the one proposed by Coomans et al. (2016),as it does not force there to be a point on the range of θ where all four response outcomes are equally likely (i.e., at δ*_i_*), but rather allows for a more flexible distribution of scores. It may be noted that the parameterization that was presented above differs from the original parameterization of Masters (1982), which would correspond to.

(7) $$\mathrm{Pr}\left(S_{i}=s,s>0|\theta \right)=\frac{\exp\sum_{r=1}^{s}\left(\theta -\beta_{\mathrm{ir}}^{*}\right)}{1+\sum_{q=1}^{3}\exp\sum_{r=1}^{q}\left(\theta -\beta_{\mathrm{ir}}^{*}\right)};$$

(8) $$\mathrm{Pr}\left(S_{i}=0|\theta \right)=\frac{1}{1+\sum_{q=1}^{3}\exp\sum_{r=1}^{q}\left(\theta -\beta_{\mathrm{ir}}^{*}\right)},$$

where $\beta_{\mathrm{is}}=-\sum_{r=1}^{s}\beta_{\mathrm{ir}}^{*}$

It can be observed that the model in equation (6) assumes all items to have the same discriminatory power. In the same way as the partial credit model and the SRT model were generalized (Muraki, 1992; van Rijn & Ali, 2018), the model in equation (6) can also be generalized to have an item‐specific discrimination parameter, denoted by α*_i_*, which allows one to differentiate the items in terms of the strength of the relationship between ability and the item score:

(9) $$\mathrm{Pr}\left(S_{i}=s|\theta \right)=\frac{\exp(s\alpha_{i}\theta +\beta_{\mathrm{is}})}{\sum_{r=0}^{3}\exp(r\alpha_{i}\theta +\beta_{\mathrm{ir}})}.$$

The larger α*_i_* is, the more information the item contains for the estimation of ability.

It should be noted that none of the models presented in equations (5), (6) and (9) generally predict a positive association between the RTs in each of the items (i.e., a positive manifold). Under these models $T_{i}^{*}$ and $T_{k}^{*}$ are expected to be positively correlated when *X_i_* = *X_k_*, but negatively correlated when $X_{i}\neq X_{k}$. The fact that they are negatively correlated when the responses differ in accuracy can be seen by contrasting the probability of the response being fast given that the response is correct and given that it is incorrect:

(10) $$\begin{array}{c} \mathrm{Pr}\left(T_{i}^{*}=1|\theta ,X_{i}=1\right)=\mathrm{Pr}\left(S_{i}=3|\theta ,S_{i}\in \left\{2,3\right\}\right)\\ =\frac{\mathrm{Pr}(S_{i}=3|\theta )}{\mathrm{Pr}\left(S_{i}=3|\theta \right)+\mathrm{Pr}\left(S_{i}=2|\theta \right)}\\ =\frac{\exp(3\alpha_{i}\theta +\beta_{i3})}{\exp\left(3\alpha_{i}\theta +\beta_{i3}\right)+\exp\left(2\alpha_{i}\theta +\beta_{i2}\right)}\\ =\frac{\exp(\alpha_{i}\theta -\beta_{i2}+\beta_{\mathrm{is}})}{1+\exp(\alpha_{i}\theta -\beta_{i2}+\beta_{\mathrm{is}})}; \end{array}$$

(11) $$\begin{array}{c} \mathrm{Pr}\left(T_{i}^{*}=1|\theta ,X_{i}=0\right)=\mathrm{Pr}\left(S_{i}=0|\theta ,S_{i}\in \left\{0,1\right\}\right)\\ =\frac{\mathrm{Pr}(S_{i}=0|\theta )}{\mathrm{Pr}\left(S_{i}=0|\theta \right)+\mathrm{Pr}\left(S_{i}=1|\theta \right)}\\ =\frac{1}{1+\exp(\alpha_{i}\theta +\beta_{i1})}\\ =\frac{\exp(-\alpha_{i}\theta -\beta_{i1})}{1+\exp(-\alpha_{i}\theta -\beta_{i1})}, \end{array}$$

where the former probability is positively related to θ while the latter is negatively related to θ (when α*_i_*> 0). Whether the unconditional correlation between a pair of RTs is positive, negative, or absent thus depends on contingent characteristics such as the item difficulties and the ability distribution, and one would not expect to consistently observe a positive manifold if an SRT model generated the data. Thus, the positive manifold that is commonly observed in RT data and that was also found in the empirical example suggests that there may be structural differences between persons in their RTs that cannot be explained by the ability parameter in the versions of the SRT model discussed so far. Therefore, we propose to further extend the model in equation (9) by including an additional latent variable that captures this possible between‐person difference in response speed.

To allow for such individual differences we extend the model in the following way:

(12) $$\mathrm{Pr}\left(S_{i}=s|\theta \right)=\frac{\exp(s\alpha_{i1}\theta_{1}+I\left(s\in \left\{0,3\right\}\right)\alpha_{i2}\theta_{2}+\beta_{\mathrm{is}})}{\sum_{r=0}^{3}\exp(r\alpha_{i1}\theta_{1}+I\left(r\in \left\{0,3\right\}\right)\alpha_{i2}\theta_{2}+\beta_{\mathrm{ir}})},$$

where θ_1_ is the ability of interest and θ_2_ is a second latent variable that accounts for individual differences in the tendency to give fast responses, α*_i_*_1_> 0 and α*_i_*_2_> 0 are the slope parameters of the item in the two dimensions,^2^ and I(s∈{0,3}) is an indicator function that takes the value 1 if the response is fast (i.e., $T_{i}^{*}=1$) and 0 if it is slow. The probability of observing values of 0 or 3 (i.e., fast responses) increases when θ_2_ increases, and hence θ_2_ can be seen as a latent speed variable. A bivariate normal distribution is assumed for the joint distribution of the two latent variables, with the means constrained to 0 and the variances constrained to 1 for identification, and a free correlation parameter ρ.

The likelihood functions for the discretized SRT models without (equation (9)) and with (equation (12)) the additional latent variable are respectively.

(13) $$L_{1}\left(\alpha ,\beta ;s\right)=\prod_{j=1}^{N}\int \prod_{i=1}^{K}\mathrm{Pr}\left(S_{i}=s_{\mathrm{ji}}|\theta ;\alpha_{i},\beta_{i}\right)N\left(\theta ;0,1\right)d\theta ,$$

(14) $$L_{2}\left(\alpha ,\beta ,\rho ;s\right)=\prod_{j=1}^{N}\int \prod_{i=1}^{K}\mathrm{Pr}\left(S_{i}=s_{\mathrm{ji}}|\theta ;\alpha_{i},\beta_{i}\right)N_{2}\left(\theta ;0,\left[\begin{array}{cc} 1 & \rho \\ \rho & 1 \end{array}\right]1\right)d\theta ,$$

where ***s*** is the *N* × *K* matrix of the observed item scores of *N* respondents on *K* items; **α** is a *K*‐vector of the item slope parameters in the unidimensional model and a *K* × 2 matrix of the item slopes in the two‐dimensional model; and **β** is a *K* × 3 matrix of the category intercept parameters of the items.

It may be noted that the proposed two‐dimensional discretized SRT model falls under the class of multidimensional nominal response models ((NRMs; Takane & De Leeuw, 1987) in the reparameterization that was proposed by Thissen and Cai (2016):

(15) $$\mathrm{Pr}\left(S_{i}=s|\theta \right)=\frac{\exp\left(\sum_{m=1}^{M}c_{m(s+1)}\alpha_{\mathrm{mi}}\theta_{m}+\beta_{\mathrm{is}}\right)}{\sum_{r=0}^{3}\exp\left(\sum_{m=1}^{M}c_{m(r+1)}\alpha_{\mathrm{mi}}\theta_{m}+\beta_{\mathrm{ir}}\right)},$$

where *M* is the number of dimensions (*M* = 2 in our case), and ***c**_m_* is a vector (in our case of length 4) containing the set of scores assigned for each possible value of *S_i_* in the *m*th dimension. For the proposed two‐dimensional discretized SRT model one would consider the item scoring functions **c**_1_ = [0,1,2,3] and **c**_2_ = [1,0,0,1] for the ability and speed dimension, respectively. While these types of multidimensional NRMs have been applied for the purpose of capturing response styles in the context of Likert scales (e.g., Falk & Cai, 2016), to our knowledge these types of models have not been considered in the context of RT modelling. A consequence of the proposed model falling into this class of multidimensional NRMs is that the model can be estimated by any software package that can deal with these types of models, such as the R package *mirt* (Chalmers, 2012).

## 5 Simulation study

### 5.1 Method

To investigate the consequences of not including the extra latent variable in the model when there are individual differences between the persons in how often they give fast or slow responses we conducted a simulation study. The focus of the study is the recovery of the ability latent variable. Two scenarios were considered: (1) a null condition, where data were generated under the unidimensional discretized SRT model (equation (9), i.e., no individual differences in RTs that cannot be explained by ability were included); (2) a condition where data were generated under the two‐dimensional model with there being individual differences in the tendency to give fast responses (equation (12)). The null condition was used as a benchmark to make sure that including the additional latent variable in the model when it is not needed does not distort the estimates of ability, and also to get benchmark values for the absolute bias, variance, and mean squared error (MSE) of the ability estimates with which the parameter recovery results when the additional dimension is present can be compared. For both scenarios, two test length conditions were considered: *K* = 20 and *K* = 40. Additionally, for scenario 2 another design factor was included, as three different values were considered for the correlation between the two latent variables, ρ = 0, .5, −.5. The values for the correlation parameter were chosen based on results from empirical studies, in which correlations close to zero (e.g., Bolsinova, Tijmstra, et al., 2017; van der Linden et al., 1999), positive correlations (e.g., van der Linden, 2007; Meng et al., 2015), and negative correlations (e.g., Klein Entink et al., 2009; van der Linden & Guo, 2008) have been found between ability and speed. In all conditions a sample size of 1,000 persons was used. For each condition, 1,000 replicated data sets were generated.

For the item threshold parameters five sets of parameters were considered: **β** *_i_* = [0,0,0], [1.5,1.5,0], [−1.5, −1.5,0], [0,1.5,1.5], [0, −1.5, −1.5].

For *K* = 20 this set was used four times, and for *K* = 40 it was used eight times. The values were chosen such that the items show reasonable differences both in their overall difficulty and in the expected proportion of slow and fast responses. For a person with zero values (i.e., average in the population) for the latent variables the five sets of threshold parameters translate into the following probabilities of the item scores: [.5,.5,.5,.5] (probability correct is equal to probability incorrect, probability fast is equal to probability slow), [.1,.4,.4,.1] (probability correct is equal to probability incorrect, probability fast is smaller than probability slow), [.4,.1,.1,.4] (probability correct is equal to probability incorrect, probability fast is greater than probability slow), [.1,.1,.4,.4] (probability correct is larger than probability incorrect, probability fast is equal to probability slow), and [.4,.4,.1,.1] (probability correct is smaller than probability incorrect, probability fast is equal to probability slow), respectively.

For the null condition, **α** *_i_* = [0.5,0] for all items, and for all other conditions **α** *_i_* = [0.5,1]. This difference between α*_i_*_1_ and α*_i_*_2_ was used because the first dimension considers scores that have a wider range (ranging from 0 to 3) than the scores considered in the second dimension (ranging from 0 to 1). On the person side, values for the two latent variables were sampled from a bivariate normal, using $\theta ∼N_{2}\left(\theta ;0,\left[\begin{array}{cc} 1 & \rho \\ \rho & 1 \end{array}\right]\right)$.

Both the unidimensional discretized SRT model (equation (9)) and the two‐dimensional discretized SRT model (equation (12)) were fitted to each replicated data set. Additionally, we fitted a hierarchical model to the replicated data sets, where 2PLMs (Birnbaum, 1968) were used to model the RAs and the discretized RTs. The hierarchical model was considered for comparison purposes, since (like the two‐dimensional discretized SRT model) it includes a speed parameter but (unlike the two‐dimensional discretized SRT model) it does not capture the relationship between RT and θ that was used to generate the data. Finally, we considered an extension of the hierarchical model which, unlike the original hierarchical model, allows for conditional dependence between RA and RT. Since RTs were discrete, we used the IRTree model for fast and slow responses (Partchev & De Boeck, 2012),, in which the item parameters in the RA model depend on whether the response is fast or slow (i.e., each item has two difficulty and two discrimination parameters in the 2PLM), while the model for RT is the same as in the hierarchical model. All models were estimated using the R package *mirt* (Chalmers, 2012) (see the Appendxi S1 and S2 for the R code used to fit the relevant models). The expected a posteriori (EAP) estimates of the person parameters were obtained given the maximum likelihood estimates of the item parameters and, in the case of the two‐dimensional models, of the correlation between the latent variables. Additionally, the EAP reliability was computed for each model.

### 5.2 Results

**Figure 2:**
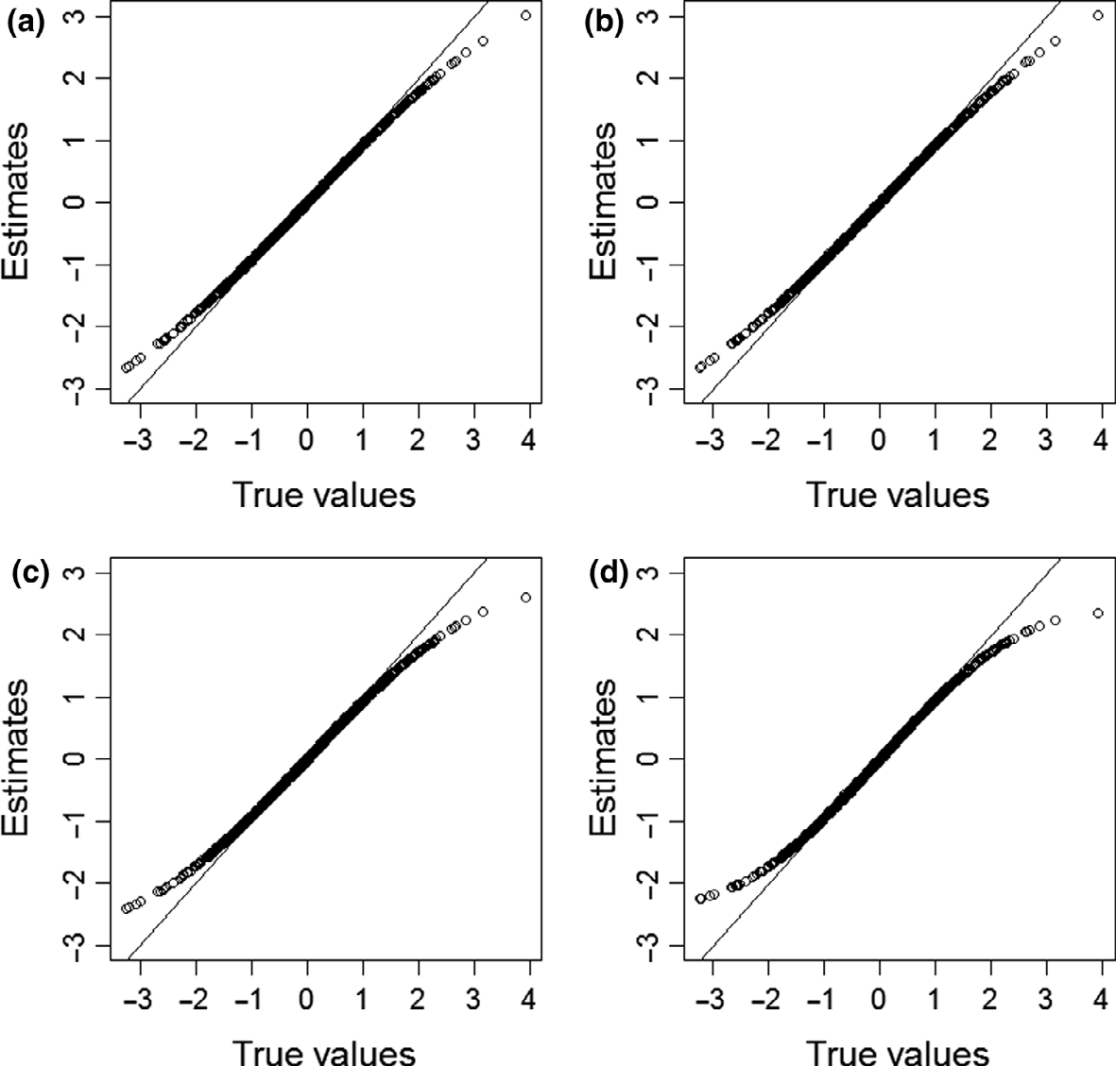
True ability parameters plotted against their average estimated value under (a) the unidimensional discretized SRT model, (b) the two‐dimensional discretized SRT model, (c) the hierarchical model, and (d) the IRTree model, obtained for 1,000 respondents based on 1,000 replications under the condition with 40 items and no person differences in speed.

The results of the simulation study are displayed in Tables 1 and 2. The first two rows of the tables show the results for the null condition (α*_i_*_2_ = 0), where it can be observed that for both *K* = 20 and *K* = 40 the absolute bias, variance, and MSE of the ability parameters are comparable for the two SRT models. Their EAP reliability is also identical. Thus, when persons do not differ in their speed, using the two‐dimensional discretized SRT model does not worsen the quality of the estimates, and overfitting does not seem to be a problem. Both SRT models outperform the hierarchical model in terms of absolute bias, variance, MSE, and EAP reliability. This can be explained by the fact that the hierarchical model does not model the patterns in the RT data correctly. The bias in the ability estimates under the IRTree model is similar to the SRT models, but its variance is worse, resulting in a higher MSE and worse EAP reliability for the IRTree model than for the two SRT models. It may be noted that for all models the average absolute bias is notably greater than 0, which is due to the estimation procedure's shrinkage of the estimates towards the population mean. Such shrinkage can be considered desirable as it reduces prediction error (see, for example, Fox, 2010). For each person, the average ability estimate under each of the four models considered is plotted against its true value in Figure 2, which illustrates this shrinkage towards the population mean.

**Table 1:** Average absolute bias (Bias), variance (Var), and mean squared error (MSE) of the estimates of θ_1_ under the discretized SRT model with and without an additional speed dimension, under the hierarchical model, and under the IRTree model, based on 1,000 replications

α*_i_*_2_	*K*	ρ	1D SRT model			2D SRT model		
			Bias	Var	MSE	Bias	Var	MSE
0	20	–	0.127	0.145	0.177	0.127	0.145	0.178
	40	–	0.067	0.088	0.099	0.067	0.089	0.099
1	20	.0	0.193	0.140	0.230	0.140	0.148	0.199
		.2	0.201	0.140	0.228	0.151	0.140	0.193
		.5	0.207	0.140	0.233	0.151	0.140	0.192
	40	.0	0.181	0.086	0.159	0.077	0.095	0.115
		.2	0.200	0.083	0.163	0.088	0.091	0.112
		.5	0.199	0.082	0.168	0.087	0.092	0.115

α*_i_*_2_	*K*	ρ	Hierarchical model			IRTree model		
			Bias	Var	MSE	Bias	Var	MSE
0	20	–	0.148	0.165	0.212	0.129	0.155	0.198
	40	–	0.077	0.105	0.122	0.066	0.098	0.117
1	20	.0	0.167	0.166	0.232	0.143	0.160	0.209
		.2	0.181	0.154	0.224	0.155	0.148	0.204
		.5	0.182	0.154	0.225	0.155	0.148	0.202
	40	.0	0.125	0.106	0.143	0.077	0.103	0.121
		.2	0.138	0.098	0.140	0.091	0.096	0.117
		.5	0.136	0.098	0.143	0.089	0.098	0.121

α*_i_*_2_ is the item slope in the second dimension (speed) of the two‐dimensional SRT model, *K* is the number of items in the test, and ρ is the correlation between the latent variables.

**Table 2:** Model‐based expected a posteriori reliability of θ_1_ under the discretized SRT model with and without an additional speed dimension, under the hierarchical model, and the IRTree model averaged across 1,000 replications

α*_i_*_2_	*K*	ρ	1D SRT	2D SRT	HM	IRTree
0	20	–	.825	.825	.791	.811
	40	–	.903	.903	.882	.892
1	20	.0	.835	.804	.789	.796
		.2	.836	.810	.798	.806
		.5	.836	.810	.798	.806
	40	.0	.908	.886	.880	.883
		.2	.913	.890	.886	.887
		.5	.914	.889	.886	.886

α*_i_*_2_ is the item slope in the second dimension (speed) of the two‐dimensional SRT model, *K* is the number of items in the test, and ρ is the correlation between the latent variables.

When persons do differ in their speed (see Table 1, α*_i_*_2_ = 1), the two SRT models show differences in performance. In all these conditions, the SRT model without the extra dimension shows notably more absolute bias in the estimated ability parameter, indicating that ignoring this secondary dimension may confound measurement. The relative difference between the two SRT models in absolute bias is bigger for the longer test (*K* = 40) than for the shorter test (*K* = 20). In contrast, the SRT model without the secondary dimension shows slightly lower variance in the estimates than the model that does include this dimension. Compared to the decrease in bias, this increase in variance is relatively small, suggesting that the small loss in precision may be outweighed by the decrease in bias. This is also apparent when the MSEs are considered, which shows the two‐dimensional SRT model to perform notably better than the one‐dimensional SRT model in all conditions where persons differ in speed. The decrease in MSE obtained when including the second dimension in the model is most notable for longer tests (*K* = 40), where a reduction of 27%–33% was observed. It should be noted that the model‐based EAP reliability is higher for the 1D SRT model than for the 2D SRT model, which can be explained by considering that, due to being misspecified, the 1D SRT model assumes the RTs to contain more information about ability than they actually do. That is, the 1D SRT model incorrectly assumes that all patterns in the RTs have to relate to ability, inflating the estimated precision and hence resulting in a higher model‐based EAP reliability than the 2D SRT model, which does not make that assumption.

Compared to the two‐dimensional SRT model, the hierarchical model shows worse performance in all conditions, in terms of both the average absolute bias and the variance in the estimates. Consequently, the hierarchical model shows a worse MSE than the two‐dimensional SRT model in all conditions, with the former having an MSE that was 24%–26% larger than that of the latter for *K* = 40. It also has a worse model‐based EAP reliability in all conditions. The hierarchical model does outperform the 1D SRT model in terms of absolute bias, which can be explained by the fact that the misspecification present in the 1D SRT model notably affects the bias of the ability estimates, whereas the misspecification present in the hierarchical model has relatively little impact on the estimates of ability since the misspecification concerns the RT side of the model, which only influences ability estimates through the correlation between speed and ability. Thus, even though both models are misspecified, the ability estimates under the hierarchical model are less severely affected.

When considering the IRTree model, the results are only slightly worse than those obtained for the two‐dimensional SRT model. For the IRTree model the absolute bias is comparable or slightly higher in each condition, and the variance is also slightly higher. Consequently, the IRTree model has somewhat worse MSE in each condition. It also shows slightly lower EAP reliability. However, the difference between the IRTree results and those of the two‐dimensional SRT model are not nearly as large as they were when considering the hierarchical model, suggesting that the IRTree model does a reasonable job of recovering the ability parameters. This can likely be explained by the fact that, unlike the hierarchical model, it allows for separate item parameters for fast and slow responses, making the model rather flexible.

All four models show a slight increase in bias when the correlation between speed and ability increases. The three models that include a speed parameter show a slight decrease in variance when this correlation increases, which can be explained by the fact that the speed parameter provides collateral information for the estimation of ability.

**Figure 3:**
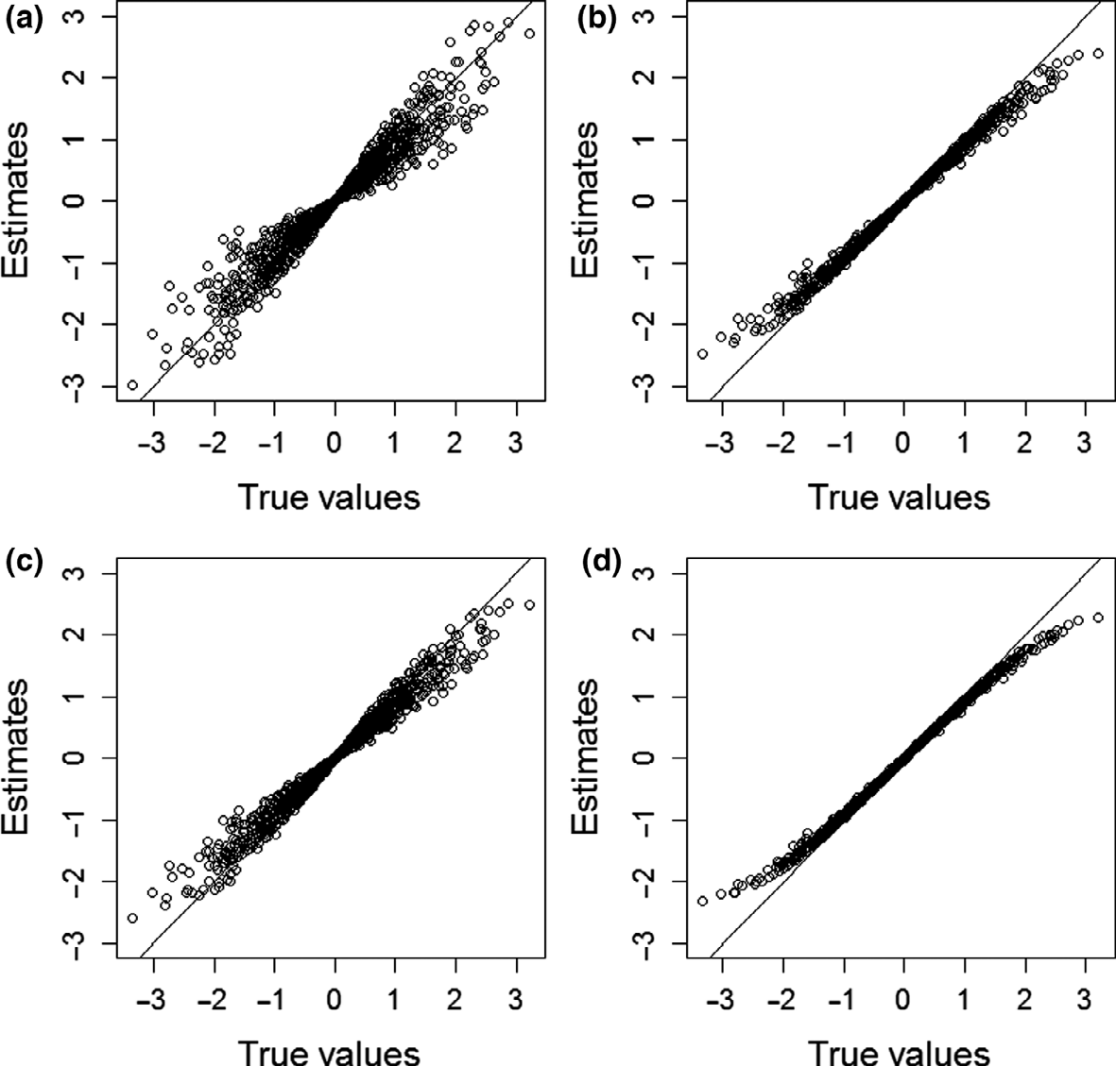
True ability parameters plotted against their average estimated value under (a) the unidimensional discretized SRT model, (b) the two‐dimensional discretized SRT model, (c) the hierarchical model, and (d) the IRTree model, obtained for 1,000 respondents based on 1,000 replications under the condition with 40 items, person differences in speed, and a correlation of 0 between the speed and ability parameter.

Figure 3 plots the true values of ability against their estimated values under the four different models, for the condition where *K* = 40 and ρ = 0 While under the unidimensional SRT model there was hardly any bias for θ_1_s close to 0, notable bias occurs for both persons with high values of θ_1_ and persons with low values of θ_1_. In contrast to what was observed under the null condition (see Figure 2), both positive and negative bias is observed for low values of θ_1_ and for high values of θ_1_, and the bias is also much more severe. When incorporating the speed parameter in the model (Figure 3b), the bias is greatly reduced compared to what was observed when using the unidimensional model (Figure 3a). Moreover, the pattern of the bias observed in Figure 3b resembles the pattern observed in Figure 2b, as both show a similar amount of shrinkage towards the mean. These results suggest that the two‐dimensional SRT model appears to be able to adequately correct for the presence of the speed factor. The results for the hierarchical model (Figure 3c) show the performance of the model in terms of bias to fall somewhere between that of the unidimensional and that of the two‐dimensional SRT model: the bias observed under the hierarchical model is less severe than what is observed under the unidimensional SRT model, but more severe than the bias observed under the two‐dimensional SRT model. For the IRTree model, the bias observed in Figure 3d is similar to that in Figure 2a, which is in line with the reported result that under the IRTree model bias is not notably worsened when person differences in response speed are introduced. While the results for the IRTree model seem similar to those obtained for the two‐dimensional SRT model, as reported in Table 1 the IRTree model shows slightly worse bias, which is most notable for extreme values of ability.

## 6 Empirical example revisited

### 6.1 Method

The same data set as presented in Section 3 was used to illustrate the use of the proposed model. Responses with RTs below 10 seconds (i.e., half of the time limit) were coded as fast, otherwise they were coded as slow. Scores were assigned to the responses using the scoring rule in equation (4).^3^ The average proportion of fast responses per item was .74, with a standard deviation of 0.04 across items, suggesting that for most respondents the item time limit was not overly restrictive. In addition to the discretization of RTs into being either fast or slow, we considered a three‐category discretization, splitting the available time into three equal intervals (below 20/3 s, between 20/3 and 40/3 s, and above 40/3 s), to check whether the model inferences change substantially if a different discretization is used. In that case, a 0, 1, 2, 3, 4, 5 coding was used for the item scores, following the same logic based on the scoring rule of the continuous SRT model.

The unidimensional and two‐dimensional SRT models were fitted to the data. Additionally, the hierarchical model and the IRTree model that were also considered in the simulation study were fitted to the data for comparison purposes. For completeness, we also fitted a unidimensional NRM to the data, with the purpose of checking whether the need to include a secondary dimension could be avoided by using a flexible unidimensional model (i.e., to ensure that any indicated need to move away from a unidimensional model is not simply due to the considered unidimensional model being too restrictive). The models were estimated using the R package *mirt* (Chalmers, 2012) using the EM algorithm.

### 6.2 Results

The results for each of the five models and for both discretization options are displayed in Table 3, as well as the results for the continuous SRT model that was fitted in Section 3. While the model fit of the continuous SRT model cannot be compared directly to that of the 1D SRT model (since the latter uses discretized versions of RT rather than continuous RT), the results for the 1D SRT model in terms of the model‐based EAP reliability suggest that measurement precision is not reduced when using discretized versions of RT rather than continuous RT. However, it should be noted that a direct comparison of model‐based EAP reliability may be misleading if either model is misspecified, which is exactly what is suggested by the results presented in Section 3.

**Table 3:** Information criteria (Akaike (AIC) and Bayesian (BIC)) and model‐based expected a posteriori (EAP) reliability for the fitted models: continuous signed residual time model (SRT), nominal response model (NRM), unidimensional and two‐dimensional discretized signed residual time models (1D SRT and 2D SRT), hierarchical model (HM), and item response tree (IRTree) model. AIC and BIC are not given for the continuous SRT because it was fitted using a Bayesian procedure

Model	AIC	BIC	EAP reliability
No discretization			
SRT			.768
Half‐deadline split			
NRM	81,065.83	82,189.05	.804
1D SRT	82,314.57	83,063.38	.773
2D SRT	78,785.01	79,727.06	.711
HM	79,677.11	80,431.96	.757
IRTree	78,788.53	79,917.80	.713
Three‐way split			
NRM	111,930.02	113,802.06	.848
1D SRT	114,784.22	115,907.44	.805
2D SRT	108,821.21	110,137.67	.708
HM	110,810.22	111,752.28	.759
IRTree	108,835.24	110,526.11	.706

The results in Table 3 allow us to assess whether the model is better able to capture the patterns observed in the data by including a second latent variable in the model. It can be observed that for both considered discretizations of RT the 2D SRT model shows better fit to the data than the 1D SRT model, since both the Akaike (AIC) and Bayesian (BIC) information criteria are notably better for the former model. This is in line with the results discussed in Section 3, where there were already strong indications that including a latent variable that explains person differences in the RTs is needed. The 2D SRT model also shows better fit than the unidimensional NRM, suggesting that it is not just the added flexibility of the 2D SRT model that allows it to have better fit than the 1D SRT model, but rather that there really is a need to include a latent variable in the model that explains person differences in the RTs. It may be noted that both the 1D SRT model and the NRM result in higher model‐based EAP reliability than the 2D SRT model, which can be considered problematic for the former models if we accept that they misrepresent the structure in the data. That is, due to the assumed unidimensionality and the item coding (combining RT and RA information in an ordered item score), both models assume RTs to be more informative of ability than the 2D SRT model suggests that they are, which would lead EAP reliability to be overestimated under the 1D SRT model and NRM.

Compared to the 2D SRT model, the hierarchical model shows worse fit, with AIC and BIC values being lower for both discretizations. Thus, the data suggest that the 2D SRT model should be preferred over the hierarchical model, and that the hierarchical model may not provide the best description of the structure in the data. While the model‐based EAP reliability is higher for the hierarchical model than for the 2D SRT model, this does not suggest that the hierarchical model should be preferred since the hierarchical model does not appear to describe the data well and hence it may not be safe to rely on the model‐based EAP reliability as an indicator of the quality of measurement under the hierarchical model.

When comparing the 2D SRT model to the IRTree model, the differences are less pronounced. For both discretizations and according to both the AIC and the BIC, the 2D SRT model should be preferred. However, the differences appear to be smaller than when contrasting the 2D SRT model with the other models. This is also in line with the simulation results reported in the previous section, where the 2D SRT model only outperformed the IRTree model by a small margin. The model‐based EAP reliability of both models is also rather similar. However, conceptually there are relevant differences between the two models that could motivate a choice between the two models, in the sense that the latent variable that relates only to the RTs has a different meaning in the two models. In the IRTree model, its interpretation matches that of the latent speed variable in the standard hierarchical model, and hence the latent variable simply captures between‐person differences in response speed. In the context of the 2D SRT model, the RTs are also included in the measurement model for ability, and the added latent variable essentially explains between‐person differences in the extent to which respondents deviate from their model‐implied response speed; that is, the latent variable may be more relevantly interpreted as the extent to which a respondent shows risk‐seeking or risk‐averse behaviour, in terms of responding faster or more slowly than is optimal under the SRT scoring rule. Since this application focuses on a setting where respondents are informed that RTs are taken into account in their score, using the 2D SRT model rather than the IRTree model might be considered more appropriate. Choosing the 2D SRT model can also be said to provide more practically relevant information for the test taker and test administrator, in the sense that this added latent variable can provide personalized insight into whether future test performance can be improved by responding more quickly or more slowly, and might also be of substantive interest in its own right (e.g., for studying whether this latent variable is indeed connected to psychological constructs such as risk seeking or risk avoidance). For these reasons we will continue investigating the results obtained for the 2D SRT model, where we will focus mainly on the half‐deadline split discretization.

**Figure 4:**
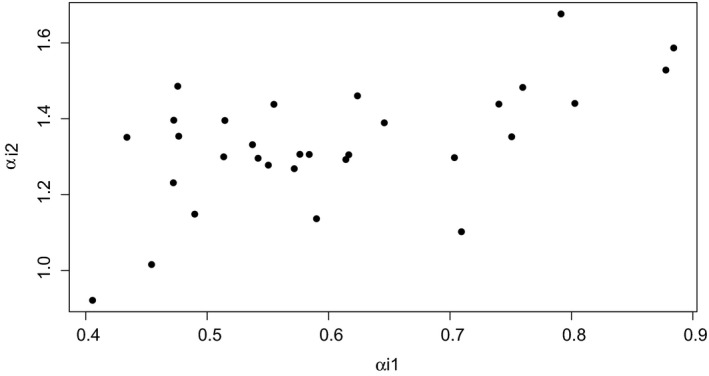
Estimates of α_1_s (on the *x*‐axis) and α_2_s (on the *y*‐axis).

On the item side, the estimates obtained for the 2D SRT model support the conclusion that there are relevant between‐person differences in response speed that can be modelled. The average value of the estimates of α_2_ was 1.33, and the estimated values for the items ranged from 0.92 to 1.68. Thus, the items discriminate persons rather well on the secondary dimension. Figure 4 plots the estimated values of α_2_ against the estimated values of α_1_ for each item, where it can be observed that there does not appear to be a clear relationship between the two parameters. Thus, it is not the case that items for which a high estimate of α_2_ was obtained are generally items that discriminate relatively well or relatively poorly on the dimension of interest (θ_1_), and hence the degree to which an item is sensitive to a person's speed does not appear to have a notable impact on its quality as an indicator of ability.

On the person side, the correlation between the two latent variables was considered. The correlation was estimated at .16, with the 95% confidence interval ranging from .11 to .22, suggesting a weak positive association between speed and ability. Thus, for the application considered, persons who respond more speedily are on average more able than those who respond relatively slowly.

**Figure 5:**
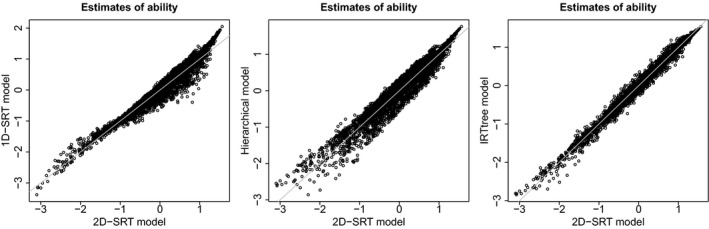
Estimates of θ under the different models for discretized response times.

The practical impact of using the 2D SRT model instead of the other models for discretized RTs can be gauged by contrasting the ability estimates obtained under the 2D SRT model with those obtained under the other models (see Figure 5). It can be observed that the estimates obtained under the two SRT models show notable differences for many of the respondents. Moreover, there appear to be slight patterns in these differences: persons of average ability (estimated θ around 0) on average get slightly higher estimates under the two‐dimensional model than under the unidimensional model, while the opposite holds for persons of high ability. Without knowledge of the generating model, it is difficult to assess which set of estimates should be preferred, and which could be investigated using further empirical research. As the current results indicate that persons do differ notably in their overall speed (as evidenced by the non‐zero item discrimination parameters obtained for the second dimension), it seems that considering a model for this application that does not include a speed latent variable may result in notable confounding of the measurement of ability.

Figure 5 also contrasts the ability estimates under the three models that include a latent speed variable. Here it can be seen that there are notable differences between the ability estimates obtained under the 2D SRT model and the hierarchical model, with differences up to 0.5 points not being uncommon. Thus, using the 2D SRT model instead of the hierarchical model might lead to substantively different conclusions about ability. The differences between the estimates obtained under the 2D SRT model and the IRTree model are notably smaller, but still present. As discussed, these models differ less in their ability to capture the patterns observed in the data, and the inferences based on these models also appear to be more similar. Compared to both the 1D SRT and the hierarchical model, these two models allow for a relatively complex relationship between RT and accuracy, which may explain why they result in relatively similar estimates of ability. However, as noted, the conceptual differences between the two models might lead one to prefer one over the other.

To study the degree to which model inferences depend on the choice of discretizing RT, we compared the results obtained using the half‐deadline split discretization (i.e., two‐category RTs) with those obtained using the three‐way split (i.e., three‐category RTs). The correlation between the ability estimates in the two‐dimensional SRT models estimated for two‐category RTs and for three‐category RTs was .987. The estimated correlation between the speed and ability latent variables was .17 in the model for the three‐category RTs. Thus, the results obtained for the two discretizations hardly seem to differ, suggesting that the particular choice of discretization of RT need not have a notable impact on the model inferences.

## 7 Discussion

In this paper a two‐dimensional discretized SRT model is considered, which maintains the idea of the traditional SRT model that RT may contain relevant information about ability, while acknowledging that persons also differ in the speed at which they take the test. By incorporating a speed latent variable, the proposed model aims both to remove the confounding effect of speed for the estimation of ability that may occur under the traditional SRT model, and to gain a more complete picture of the respondents and the way in which they take the test. That is, the model provides information not only about ability, but also about the speed at which a person takes the test. This latter latent variable captures associations between RTs that are not explained by ability. As such, one can expect it to be related to psychological attributes such as response caution and risk seeking. However, the exact interpretation of this latent variable will likely depend on the particular application that is considered, and empirical research would be needed to validate any claim about this latent variable capturing a particular response tendency such as response caution.

The proposed model makes use of dichotomized RT rather than continuous RT, and as such does not have the standard SRT model as a special case. The dichotomization was considered to enable the modelling of the speed latent variable using the toolbox of multidimensional polytomous IRT. For this paper, a dichotomization at the middle of the theoretical range of RT was used, and an item scoring motivated based on the SRT model for continuous RT was implemented, in line with the scoring rule of Coomans et al. (2016). However, depending on the application considered, it may make sense to use a different dichotomization of RT. Care should be taken that using data‐dependent thresholds (such as those based on any form of median split) may be at odds with the idea that a scoring rule should be determined a priori and communicated to the respondents.

While different dichotomizations of RT can be considered, it may be emphasized that if one wants to maintain a scoring rule that is based on the SRT model with continuous RT, the choice of dichotomization should be reflected in the scoring rule. An alternative could be to depart from the scoring rule suggested by the standard SRT model, for example if one feels that the difference between a fast and a slow correct response should be seen as less (or more) indicative of an ability difference than the difference between a slow correct and a slow incorrect response. Ultimately, the choice of the scoring rule should be subjected to validation research if the purpose is to obtain unconfounded measurement of a particular ability.

In addition to employing different dichotomizations of RT, one can consider using different ways of discretizing RT, for example by binning it in three or more categories (e.g., slow, medium, fast), as was also considered in the context of the empirical example. For such a polytomous categorization of RT one would need to carefully consider the scores that are assigned to each combination of RA and RT for both the speed and the ability dimension, but such an approach might allow for finer‐grained analysis of the response processes. Conversely, further study of the response processes might result in a more informed decision on how to discretize RT and how to score the response outcomes in the most meaningful or relevant way. However, care should also be taken to avoid issues with sparsity, which may arise if RTs are binned in more than a few categories.

It may be argued that in high‐stakes testing settings, whatever scoring rule is adopted and communicated to the respondents should be maintained, and hence that it is up to the respondents to optimize their test‐taking behaviour in accordance with this scoring rule. In this sense the respondent who shows notable response caution on a test where the SRT scoring rule is adopted is simply using a suboptimal testing strategy for which perhaps no correction should be implemented. As such, it may not necessarily be appealing to employ the two‐dimensional discretized SRT model when dealing with a high‐stakes test. However, for low‐stakes testing settings the main purpose is commonly to obtain as accurate as possible a picture of the true ability of each respondent, and correcting for distorting factors such as differences in response caution can be considered desirable. Thus, the proposed model is likely to be of most use in low‐stakes testing settings, such as the application considered in the empirical example. Additionally, using the two‐dimensional model in low‐stakes practice settings could be very useful for providing test takers with feedback on the extent to which their response speed deviates from the model‐implied optimal response speed, with persons who have an extreme value on this second latent variable likely benefiting from altering their test‐taking behaviour. This kind of feedback could be helpful if the same test taker will later be faced with a high‐stakes test where performance is evaluated based on the SRT scoring rule.

## 8 Conflicts if interest

All authors declare no conflict of interest.

## Author contributions

Jesper Tijmstra, Ph.D. (Conceptualization; Formal analysis; Investigation; Methodology; Project administration; Resources; Software; Writing – original draft; Writing – review & editing) Maria Bolsinova (Conceptualization; Data curation; Formal analysis; Investigation; Methodology; Software; Visualization; Writing – original draft; Writing – review & editing).

## Supporting information

**Appendix S1.** This Appendix contains the R code for the simulation study and the analysis of the empirical example presented in this paper.

## Data Availability

Access to data can be acquired by contacting the second author.

## Footnotes

^1^
Independent improper priors were used for the item parameters. The person abilities were assumed to have a standard normal distribution. Data augmentation was implemented, such that at each iteration individual ability parameters were sampled for each person using single‐variable exchange algorithm (Marsman, 2014). Discrimination and difficulty parameters were sampled using a Metropolis algorithm with a (log)normal distribution centred around the (log of the) current value.

^2^
Both item slope parameters should be positive, to make sure that *S_i_* is positively related to θ*_i_* and that being fast is positively related to θ_2_, respectively.

^3^
Note that if responses are possible after the time limit expires and if this occurs with a non‐negligible frequency (which was not the case in this example), the scoring rule can be adapted as follows: 0, fast incorrect responses; 1, slow incorrect responses within the time limit; 2, responses after the time limit; 3, slow correct responses within the time limit; 4, fast correct responses.
